# O5-6 The relation between domain-specific physical behavior and cardiorespiratory fitness: a compositional data analysis on the physical activity health paradox using accelerometer-assessed data

**DOI:** 10.1093/eurpub/ckac094.038

**Published:** 2022-08-29

**Authors:** Bart Cillekens, Margo Ketels, David Hallman, Nidhi Gupta, Els Clays, Huysmans Maaike, Holtermann Andreas, Pieter Coenen

**Affiliations:** 1 Department of Public and Occupational Health, AmsterdamUMC, Amsterdam, The Netherlands; 2 Department of Public Health and Primary Care, Ghent University, Gent, Belgium; 3 Department of Occupational and Public Health Sciences, University of Gävle, Gavle, Sweden; 4 National Research Centre for the Working Environment, National Research Centre for the Working Environment, Copenhagen, Denmark

**Keywords:** Fitness, Occupational physical activity, PA-domains, device based measurements

## Abstract

**Background:**

In contrast to leisure time physical activity (LTPA), occupational physical activity (OPA) does not have similar beneficial health effects. These differential health effects, also known as the physical activity health paradox, might be explained by dissimilar effects of LTPA and OPA on cardiorespiratory fitness (CRF). This study aims to investigate the association between device-worn measures of physical behaviors during both work and leisure time and CRF among workers with high level of OPA.

**Methods:**

Our results are based on a sample of 309 workers employed within the service and production sector from the cross-sectional FEPA (Flemish Employees' Physical Activity) study. OPA and LTPA were measured using two Axivity AX3 accelerometers, worn on the back and right thigh for 2 to 4 consecutive working days. CRF levels were obtained by the Harvard step test. Compositional multiple linear regression analyses were used to analyze the relations, adjusted for age, sex, education, smoking, BMI, moderate-to vigorous physical activity (MVPA), and physical work demands.

**Results:**

During work time, more sedentary behavior (SB) was associated with higher CRF when compared relatively to time spent on other work behaviors, while more SB during leisure time was associated with lower CRF when compared to other leisure time behaviors. Reallocating more time to MVPA from the other behaviors within leisure time was positively associated with CRF, which was not the case for MVPA during work.

**Conclusion:**

Our results emphasize the need for taking the domain-specific nature of physical activity into account to understand its relation to CRF. Guidelines usually do not differentiate between OPA and LTPA in their recommendation to participate in at least 150 min of physical activity per week, regardless of the OPA level. Workers already meeting this recommendations through OPA might therefore mistakenly think that they already meet the recommendations on physical activity and think they can spend their leisure time in a sedentary fashion. In reality, these types of workers might benefit from recommendations to take more sitting breaks during their work and to participate in leisure time MVPA to maintain or improve their CRF in order ‘to be fit for the job'.

